# Increased height standard deviation scores in response to growth hormone therapy to near-adult height in older children with delayed skeletal maturation: results from the ANSWER Program

**DOI:** 10.1186/1687-9856-2015-1

**Published:** 2015-01-15

**Authors:** Judith L Ross, Peter A Lee, Robert Gut, John Germak

**Affiliations:** Department of Pediatrics, Thomas Jefferson University, Philadelphia, PA 19107 USA; Nemours/AI DuPont Hospital for Children, Wilmington, DE 19803 USA; Department of Pediatrics, Penn State College of Medicine, The Milton S. Hershey Medical Center, Hershey, PA 17033 USA; Department of Clinical Development, Medical and Regulatory Affairs, Novo Nordisk, Inc., 800 Scudders Mill Road, Plainsboro, NJ 08536 USA

**Keywords:** Human growth hormone, Short stature, Near-adult height

## Abstract

**Background:**

A primary goal of recombinant human growth hormone therapy (GHT) in children is attaining normal adult height. In this study, children with growth hormone deficiency (GHD) (including isolated idiopathic growth hormone deficiency [IGHD] and multiple pituitary hormone deficiency [MPHD]), idiopathic short stature (ISS), and Turner syndrome (TS) were evaluated for near-adult height (NAH) and percent achieving NAH within the normal range after approximately 4 years of GHT.

**Methods:**

Data from the American Norditropin® Studies: Web-Enabled Research (ANSWER) Program were analyzed for NAH from age at treatment start (ATS) (i.e., referral age as defined by age at enrollment in the study) to last clinic visit using one of the following two criteria: 1) age ≥18 years, or 2) if male: ≥16 years and height velocity (HV) <2 cm/year; if female: ≥15 years and HV <2 cm/year. All patients had a baseline height standard deviation score (HSDS) ≤ -2, and either GHD (n = 201), ISS (n = 19), or TS (n = 41). The main outcome measures included HSDS and corrected HSDS (HSDS-target HSDS) in response to GH treatment, and correlation of ATS with NAH HSDS.

**Results:**

Mean (± SD) chronological and bone ages at baseline were 14.0 ± 2.1 years and 11.7 ± 2.0 years, respectively, and mean GHT duration was 4.0 ± 1.6 years. Mean HSDS (baseline to NAH; GHD: -2.7 to -1.0; ISS: -2.8 to -1.4; TS: -3.0 to -1.8) and mean corrected HSDS (baseline to NAH; GHD: -2.1 to -0.3; ISS: -2.1 to -0.6; TS: -1.8 to -0.6) increased across diagnostic indications. Percentages of patients reaching near-adult HSDS > -2 were GHD: 87.6%; ISS: 78.9%; TS: 65.8%. Significant negative correlations were found between ATS and NAH HSDS when analyzed by sex.

**Conclusions:**

Despite a relatively advanced childhood age, the majority of GH-treated patients attained mean near-adult HSDS within the normal range (HSDS > -2). Negative correlations of ATS with near-adult HSDS indicate that an earlier age at treatment start would likely have resulted in greater adult height achieved in both male and female patients.

## Background

Treatment with recombinant human growth hormone (GH) is indicated for children with short stature or growth failure associated with a number of conditions in which there is a deficiency of, or decreased responsiveness to, endogenous growth hormone. The use of GH is approved by the US Food and Drug Administration for the treatment of growth failure associated with growth hormone deficiency (GHD), either isolated (IGHD) or part of multiple pituitary hormone deficiency (MPHD), and short stature associated with Turner syndrome (TS), Noonan syndrome (NS), children born small for gestational age (SGA), short stature homeobox (SHOX) gene haploinsufficiency, Prader-Willi syndrome (PWS), chronic kidney disease (CKD), or idiopathic short stature (ISS) [1]. Treatment with GH has been shown to increase short-term linear growth in children with various disorders associated with growth failure; however, some of the short-term clinical studies have shown varying treatment outcomes [2–4]. A limited number of studies have investigated the effects of long-term GH therapy (GHT) on the adult height standard deviation score (HSDS) achieved and whether the attained adult height was within the normal range.

Attaining adult height within the normal range is an important treatment goal for GHT in children with short stature or growth failure disorders, especially with respect to the potential benefits in health-related quality of life (HRQoL) and physical and psychosocial well-being [5, 6]. The adult height of patients treated with GH is influenced by the height gained during the course of therapy, the rate of bone maturation, and the onset of puberty [7, 8]. Longitudinal analyses of large data sets of pediatric patients have consistently demonstrated that the early initiation and appropriate duration of GH treatment correlated significantly with the achievement of near-normal adult height and greater improvements in HSDS [2, 9, 10]. A multicenter, randomized, double-blind clinical study of children with short stature who were born SGA without signs of catch-up growth showed that GH treatment to adult height was associated with a significant improvement in HRQoL and the normalization of final height [5].

The American Norditropin Studies: Web-Enabled Research (ANSWER) Program (utilizing NovoNet®, the Novo Nordisk Web-based research platform) is an observational, noninterventional study evaluating the long-term effectiveness and safety of Norditropin (somatropin [recombinant DNA origin] injection, Novo Nordisk A/S, Bagsvaerd, Denmark), hereafter referred to as GH, therapy in pediatric and adult patients [11]. Enrollment in this patient registry is solely at the discretion of the participating physicians in patients for whom Norditropin® is prescribed for treatment of appropriate conditions of growth failure and short stature both within and outside the Norditropin® label. Data from the ANSWER Program have shown that children with IGHD, MPHD, ISS, TS, NS, or SGA demonstrated an increase in HSDS from baseline following 2 years of GH treatment [12, 13]. Height velocity (HV) at 4 months of GHT and baseline body mass index (BMI) SDS were significant predictive factors in patients with GHD (including patients with IGHD and MPHD) that positively correlated with a change in HSDS (ΔHSDS) [13]. Baseline age, baseline HSDS, and baseline serum insulin-like growth factor-1 (IGF-I) SDS were negatively correlated with ΔHSDS [12, 13]. No consistent effect of the sex of a patient on response to GH treatment was observed in these analyses from the ANSWER Program; however, a sex-related effect on ΔHSDS was reported by Savendahl et al. [14] in a combined analysis of short-term (2-year) GH treatment responses in children with GHD, MPHD, or SGA enrolled in the ANSWER Program and its European counterpart, the NordiNet® International Outcome Study. Greater growth responses were observed in younger, prepubertal children in all diagnostic groups studied in a report from the ANSWER Program, indicating that early initiation of GH treatment is important for optimizing linear growth response [12].

The principal aim of the current analysis of data from the ANSWER Program registry was to evaluate the effects of GH treatment in previously untreated pediatric patients with GHD, ISS, or TS with baseline HSDS ≤ -2 on near-adult height (NAH) outcomes. The effect of age at the start of treatment on attainment of NAH was also evaluated. NAH was evaluated only at the last clinic visit when GH was discontinued, whereas HSDS was determined at year 1, year 2, and at NAH (i.e., the last clinic visit).

## Methods

### Study design

The ANSWER Program is a non-interventional, observational study initiated in 2002 that has collected long-term effectiveness and safety information on pediatric patients treated with GH. Physicians participating in the ANSWER Program provide updated patient histories and physical examination data utilizing NovoNet®, an online data-reporting tool. Institutional review board (IRB) approval and patient informed consent are obtained before study enrollment, which is at the discretion of participating physician investigators. Pediatric patients were included in the current analyses if they were aged <18 years and GHT-naïve at the time of enrollment, had a baseline HSDS that met criteria for short stature (≤ - 2), and had attained adult height or NAH at their final clinic visit. Height velocity was used as a criterion for near adult height for boys between 16 (inclusive) and 18 years of age and girls between 15 (inclusive) and 18 years of age. In order to calculate height velocity, a height measurement had to have been taken between the minimum interval of 273 and the maximum interval of 548 days before the last visit date. The previous height assessment nearest to 1 year (365 days) prior to the last visit date was used. Height velocity was annualized by calculating the change in height per day and multiplying by 365.25 days. Patients were determined to have reached NAH according to either one of the following two criteria: 1) age at last visit was ≥18 years, or 2) for males <18 years, if their age at last visit was ≥16 years and HV <2 cm/year; or for females < 18 years, if their age at last visit was ≥15 years and HV <2 cm/year. The rationale for using the first NAH criterion of ≥18 years of age was to capture patients who had a limited frequency of clinic visits prior to the last clinic visit that prevented calculation of HV but whose age would be consistent with completion or near completion of statural growth. Patients identified using HV and age criteria had sufficient visit frequency from which to assess HV consistent with NAH. As noted in previous publications from the ANSWER Program pediatric patients are generally excluded from analysis if baseline chronologic age (CA) was <1 year or >18 years, or if baseline values of key variables were missing or deemed inconsistent or implausible (e.g., lack of height information at baseline; baseline height <35 cm or >200 cm; baseline HSDS < -5 or > +2) [13, 15]. The current study assessed children with GHD (patients with IGHD and MPHD), ISS, and TS who reached NAH after treatment with GH. Data were collected from clinic visits within a ±3-month window around each time point (i.e., year 1, year 2, NAH [i.e., last visit]) and growth rates were annualized based on the actual interval between measurements.

### Baseline characteristics and measurement of GH treatment effects

Baseline demographics and patient characteristics across all indications in GH-naïve patients were obtained just prior to the start of GH treatment (i.e., at time of enrollment in the registry). Baseline variables at enrollment included sex, CA, bone age (BA), body weight, and height [16]. Other baseline measurements were BA/CA ratio, HSDS, target HSDS, corrected HSDS (HSDS minus target HSDS), predicted HSDS based on predicted adult height calculated using the Bayley-Pinneau method [17], serum levels of IGF-I (ng/mL) and determination of IGF-I SDS, peak stimulated serum GH concentration (ng/mL), BMI (kg/m^2^), BMI SDS, and initial GH dose (mg/kg/day). At subsequent annual clinic visits, GH dose was reported along with measurements of height, weight, CA, BA, serum IGF-I concentration, and BMI.

### Variables and statistical analyses

The following key variables were determined at year 1, year 2, and NAH (i.e., last clinic visit): mean HSDS, mean corrected HSDS, mean change in HSDS (ΔHSDS), BA/CA ratio, IGF-I SDS, and BMI SDS. The percentage of patients who achieved a HSDS at NAH within the normal range (HSDS > -2) was also determined. Height SDS and BMI SDS were calculated using standard reference data developed by the United States Centers for Disease Control and Prevention [18]. Baseline demographic characteristics were summarized using descriptive statistics. Target height was evaluated using the mean height of the patient’s two parents plus 6.5 cm for male patients or minus 6.5 cm for female patients [19]. Corrected HSDS was calculated as the difference between HSDS determined at a given time point and the target HSDS. ΔHSDS was calculated as the difference between the HSDS at baseline and at year 1, year 2, and NAH. All IGF-I values were measured locally and entered into the database. Transformation of IGF-I data into IGF-I SDS was based on the age- and sex-related normative reference values of Brabant *et al*. [20]. The correlation between age at GH treatment start (ATS) and near-adult HSDS was evaluated by estimating Pearson’s correlation coefficient (R) between the 2 variables. Near-adult HSDS was analyzed using an analysis of covariance model (ANCOVA) with sex as the fixed effect and referral age as the covariate. A regression model was applied to analyze the relationship between ATS (i.e., referral age as defined by age at enrollment in the study) and near-adult HSDS for all patients combined with GHD, ISS, and TS according to sex. Values are reported as mean and standard deviation (±SD) for descriptive statistics or mean and standard error (±SE) for comparative statistics. All differences detected from statistical analyses were considered significant at *P* values <0.05.

## Results

### Patient demographics and baseline characteristics

A total of 261 GH-naïve patients who were diagnosed with GHD (n = 201), ISS (n = 19), or TS (n = 41), and had achieved NAH, were included in the analyses. This total patient number consisted of 178 patients identified according to the age criterion (≥18 years) and 83 patients identified according to age and HV criteria for NAH (20 males, 63 females). Demographics and baseline characteristics for the overall study population and for each diagnostic indication cohort are presented in Table 1. Overall, the study population consisted of 173 males (66.3%) and 88 females (33.7%), including 41 females with TS. The mean referral age was 14.7 ± 1.6 years among males and 12.8 ± 2.3 years among females. The mean ± SD baseline CA across all indications (N = 261) was 14.0 ± 2.1 years (14.9 ± 1.5 years among patients identified according to the age criterion, 12.7 ± 1.9 years among male patients identified according to the HV criterion, and 12.2 ± 2.1 years among female patients identified according to the HV criterion). Mean ± SD BA across indications at baseline was 11.7 ± 2.0 years (N = 215), approximately 2 years behind CA; this was also reflected in a baseline BA/CA ratio <1 across all patient cohorts (0.85 ± 0.08, N = 215).

**Table 1:** **Baseline demographics, characteristics, and GH treatment duration**

Characteristic	Overall		GHD		ISS		TS	
	(N = 261)		(n = 201)		(n = 19)		(n = 41)	
	N	Mean ± SD	N	Mean ± SD	N	Mean ± SD	N	Mean ± SD
Sex, M/F	173/88		158/43		15/4		0/41	
Chronologic age (CA), y								
All NAH criteria	261	14.0 ± 2.1	201	14.2 ± 1.9	19	14.0 ± 1.9	41	13.1 ± 2.5
age >18 y	178	14.9 ± 1.5	149	14.9 ± 1.5	13	14.6 ± 1.4	16	14.9 ± 2.2
male, age >16 y,	20	12.7 ± 1.9	18	12.5 ± 1.9	2	14.6 ± 0.06	-	-
HV < 2 cm/y								
female, age >15 y, HV <2 cm/y	63	12.2 ± 2.1	34	12.4 ± 2.1	4	11.9 ± 2.3	25	11.9 ± 2.0
Bone age (BA), y	215	11.7 ± 2.0	169	11.9 ± 1.9	16	11.6 ± 2.2	30	11.0 ± 2.2
BA/CA ratio	215	0.85 ± 0.08	169	0.85 ± 0.08	16	0.84 ± 0.08	30	0.84 ± 0.09
HSDS								
All NAH criteria	261	-2.8 ± 0.6	201	-2.7 ± 0.5	19	-2.8 ± 0.6	41	-3.0 ± 0.6
age >18 y	178	-2.8 ± 0.6	149	-2.7 ± 0.6	13	-2.9 ± 0.7	16	-2.9 ± 0.7
male, age >16 y, HV < 2 cm/y	20	-2.5 ± 0.4	18	-2.5 ± 0.4	2	-2.6 ± 0.0	-	-
female, age >15 y, HV <2 cm/y	63	-2.8 ± 0.5	34	-2.7 ± 0.5	4	-2.6 ± 0.3	25	-3.0 ± 0.6
Target HSDS	222	-0.3 ± 0.8	171	-0.3 ± 0.8	18	-0.7 ± 0.7	33	0.2 ± 0.9
Corrected HSDS	221	-2.1 ± 4.1	169	-2.1 ± 4.0	18	-2.1 ± 0.7	34	-1.8 ± 5.3
Predicted HSDS*	186	-1.4 ± 1.1	143	-1.2 ± 1.0	13	-1.6 ± 1.1	30	-2.1 ± 0.9
IGF-I SDS	187	-2.6 ± 1.60	152	-2.7 ± 1.60	12	-2.3 ± 1.81	23	-1.7 ± 1.22
BMI, kg/m^2^	260	19.9 ± 4.5	201	19.7 ± 4.5	18	18.1 ± 2.8	41	21.4 ± 4.9
BMI SDS	260	-0.21 ± 1.38	201	-0.32 ± 1.44	18	-0.68 ± 1.05	41	0.53 ± 0.90
Peak GH, ng/mL	166	6.9 ± 5.7	148	5.5 ± 2.8	14	20.3 ± 9.7	4	10.9 ± 8.7
Initial GH dose, mg/kg/d	259	0.048 ± 0.012	199	0.047 ± 0.013	19	0.050 ± 0.006	41	0.052 ± 0.008
Treatment duration, y	261	4.0 ± 1.6	201	3.9 ± 1.5	19	3.9 ± 1.5	41	4.4 ± 2.0

*Abbreviations*: *BMI* body mass index, *GH* growth hormone, *GHD* growth hormone deficiency, *HSDS* height standard deviation score, *IGF-I* insulin-like growth factor-1, *ISS* idiopathic short stature, *M/F* male/female, *TS* Turner syndrome, *SDS* standard deviation score; y, years.

Predicted HSDS was calculated by the Bayley-Pinneau method [17] using a child’s current height, sex, chronological age, and bone age.

At baseline (Table 1), the mean ± SD stimulated peak serum GH concentration (ng/mL) was 5.5 ± 2.8 in patients with GHD, 20.3 ± 9.7 in patients with ISS, and 10.9 ± 8.7 in females with TS. Mean ± SD IGF-I SDS was -2.7 ± 1.60 in patients with GHD, -2.3 ± 1.81 in those with ISS, and -1.7 ± 1.22 in females with TS.

The mean ± SD duration in years of GH treatment by indication (Table 1) was 3.9 ± 1.5 for both GHD and ISS groups, and 4.4 ± 2.0 in females with TS. The mean initial GH doses (mg/kg/d) administered to each patient cohort at treatment start (Table 1) were 0.047 ± 0.013 (n = 199) in patients with GHD, 0.050 ± 0.006 (n = 19) in those with ISS, and 0.052 ± 0.008 (n = 41) in females with TS.

### Analysis of auxological measurements

#### Height standard deviation scores

**Figure 1:**
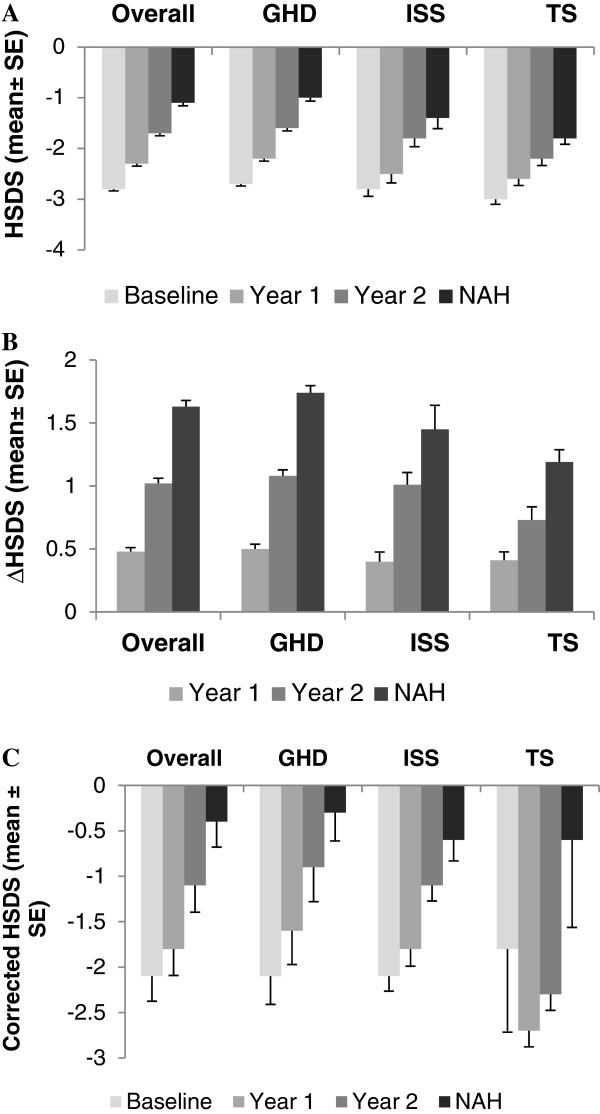
**HSDS, ΔHSDS and corrected HSDS in patients from baseline to near-adult height at the last visit. (A)** Mean HSDS at baseline, year 1, year 2, and NAH at the last clinic visit. **(B)** Mean ΔHSDS (HSDS-target HSDS) at year 1, year 2, and NAH at the last visit. **(C)** Mean corrected HSDS at baseline, year 1, year 2, and NAH at the last visit. GHD: growth hormone deficiency; HSDS: height standard deviation score; ISS: idiopathic short stature; NAH: near-adult height; TS: Turner syndrome.

The mean ± SD baseline HSDS was -3.0 ± 0.6 (n = 41) for patients with TS, -2.8 ± 0.6 (n = 19) for patients with ISS, and -2.7 ± 0.5 (n = 201) for patients with GHD. Target HSDS (mean ± SD) at baseline was -0.7 ± 0.7 (n = 18) for patients with ISS, -0.3 ± 0.8 (n = 171) for patients with GHD, and +0.2 ± 0.9 (n = 33) for patients with TS (Table 1). Mean ± SE HSDS gradually increased over time for the overall study population from -2.8 ± 0.04 (n = 261) at baseline to -1.1 ± 0.06 (n = 261) at NAH (Figure 1A). Patients with GHD, ISS, and TS experienced increases in mean ± SE HSDS from baseline to NAH (near-adult HSDS: GHD, -1.0 ± 0.065, n = 201; ISS, -1.4 ± 0.210, n = 19; TS, -1.8 ± 0.118, n = 41). Both mean ± SE ΔHSDS (Figure 1B) and mean ± SE corrected HSDS (Figure 1C) increased with GH treatment (ΔHSDS at NAH: GHD, 1.74 ± 0.06, n = 201; ISS, 1.45 ± 0.19, n = 19; TS, 1.19 ± 0.10, n = 41; baseline corrected HSDS: GHD, -2.1 ± 0.31, n = 169; ISS, -2.1 ± 0.16, n = 18; TS, -1.8 ± 0.92, n = 34; corrected HSDS at NAH: GHD, -0.3 ± 0.31, n = 169; ISS, -0.6 ± 0.23, n = 18; TS, -0.6 ± 0.96, n = 34). The mean ± SD GH dose (mg/kg/d) at NAH for each cohort across indications (GHD: 0.050 ± 0.018, n = 199; ISS: 0.052 ± 0.012, n = 19; and TS: 0.053 ± 0.011, n = 41) had not changed markedly from treatment start. The majority of patients in each indication achieved heights in the normal range (> - 2 HSDS) by the last visit: 87.6%, 78.9%, and 65.8% of GHD, ISS and TS patients, respectively (83.5% of patients overall).

#### Age at treatment start and near-adult HSDS

**Figure 2:**
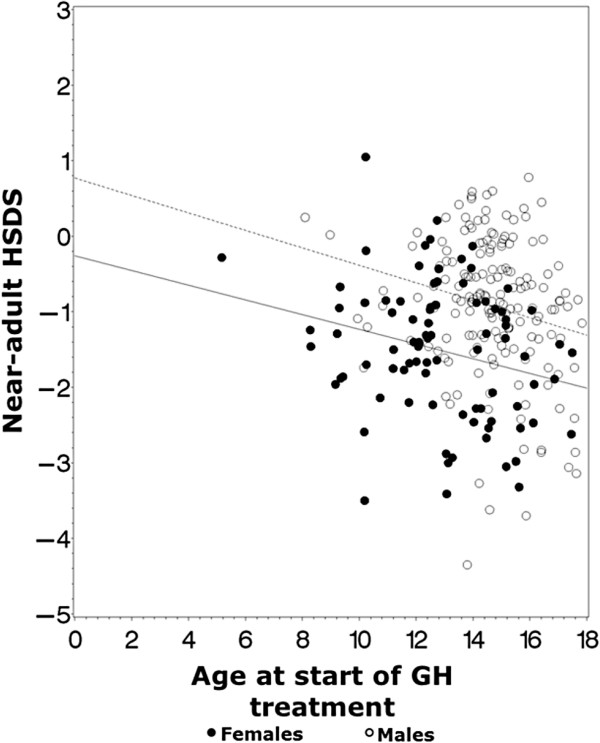
**Effects of age at treatment start on adult HSDS by sex.** Correlation between ATS and adult HSDS (*P* < 0.001). The effects of ATS on adult HSDS vary depending on sex (*P* < 0.001; ANCOVA). ATS: age at treatment start; HSDS: height standard deviation score. Top line: Regression of near-adult HSDS against ATS for males. Bottom line: Regression of near-adult HSDS against ATS for females.

When evaluating the total patient population, no significant correlation was found between ATS and near-adult HSDS (Pearson’s correlation coefficient, R = -0.072, *P* = 0.25). The ANCOVA model with sex as the fixed effect and referral age as the covariate confirmed that sex (*P* < 0.001) and referral age (*P* < 0.001) were statistically significant, indicating a relationship between near-adult HSDS, referral age and sex. When analyzing this relationship separately by patient sex, significant negative correlations were found for both males and females (including patients with TS): males, Pearson’s correlation coefficient, R = -0.21, *P =* 0.006; females, Pearson’s correlation coefficient, R = -0.25, *P* = 0.017 (Figure 2).

#### IGF-I and BMI

**Figure 3:**
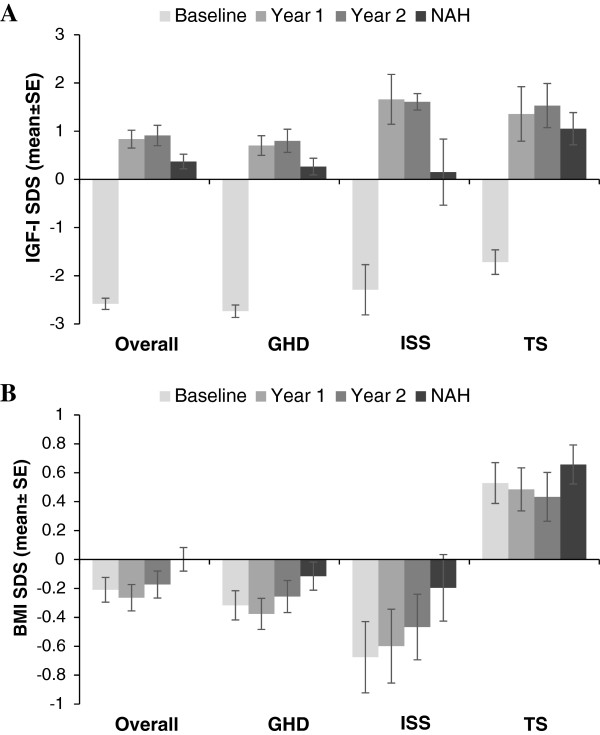
**IGF-I SDS and BMI SDS from baseline to near-adult height at the last visit. (A)** IGF-I SDS at baseline, year 1, year 2, and NAH at the last clinic visit. **(B)** BMI SDS at baseline, year 1, year 2, and NAH at the last visit. BMI: body mass index; GHD: growth hormone deficiency; IGF-1: insulin-like growth factor-1; ISS: idiopathic short stature; NAH: near-adult height; SDS: standard deviation score; TS: Turner syndrome. Error bars represent standard error (SE).

The attainment of NAH across patient cohorts was paralleled by increases in mean ± SE IGF-I SDS (Figure 3A) to within the normal reference range across all indications (GHD at baseline: -2.74 ± 0.130, n = 152; GHD at NAH: 0.27 ± 0.172, n = 193; ISS at baseline: -2.29 ± 0.522, n = 12; ISS at NAH: 0.15 ± 0.687, n = 16; TS at baseline: -1.72 ± 0.255, n = 23; TS at NAH, 1.05 ± 0.333, n = 35). Mean ± SE BMI SDS (Figure 3B) showed minimal change for each indication over time during GH treatment (GHD at baseline: -0.32 ± 0.101, n = 201; GHD at NAH: -0.11 ± 0.097, n = 201; ISS at baseline: -0.68 ± 0.246, n = 18; ISS at NAH: -0.20 ± 0.230, n = 19; TS at baseline: 0.53 ± 0.141, n = 41; TS at NAH: 0.66 ± 0.135, n = 41). Mean ± SE BA/CA ratio remained stable over time during GH treatment across all indications (GHD at baseline: 0.85 ± 0.006, n = 169; GHD at NAH: 0.86 ± 0.006, n = 197; ISS at baseline: 0.84 ± 0.019, n = 16; ISS at NAH: 0.88 ± 0.021, n = 19; TS at baseline: 0.84 ± 0.016, n = 30; TS at NAH: 0.82 ± 0.014, n = 39).

## Discussion

The results of this analysis of GH treatment in pediatric patients with GHD, ISS, or TS demonstrated that GH-naïve children with short stature achieved a mean NAH within the normal reference range. Mean HSDS, corrected HSDS, and IGF-I SDS all improved over time with GH treatment, and the majority of patients were within the normal reference range (> - 2 HSDS) at the time of NAH. Overall, 83.5% of the study population had attained normal height (HSDS > -2) over the duration of GH treatment (mean duration, years: GHD 3.9; ISS 3.9; TS 4.4). It is possible that the results were biased towards good responders, since poor responders may have withdrawn from the study or stopped GH treatment before reaching adulthood. The proportion of TS patients who attained normal height (66%) in this study was smaller compared with those of the other indications studied (79-88%). The reasons for this finding are unclear; nevertheless, this result is consistent with findings from a previous analysis of patients with TS from the ANSWER program [21].

At baseline, the mean ± SD overall age was 14.0 ± 2.1 years (12.8 years in females and 14.7 years in males), which was nearly 4 years older than the mean age of pediatric patients typically enrolled in the ANSWER Program (10.5 ± 4.0 years). Consequently, this analysis of patients who attained NAH was skewed toward children who were older at the time of GHT initiation, and thus may not be representative of the overall population in the ANSWER Program. The short duration of the Program (initiated in 2002) could have contributed to a selection bias towards older patients since a substantial proportion of enrolled patients would not have reached 18 years as of the cut-off date for this current analysis. The older age at baseline in this subgroup may have resulted in an underestimation of the overall adult height achievable by all patients treated with GH. Previous studies from the ANSWER Program have shown that earlier age at GH treatment initiation is associated with greater height gain [12, 13], reinforcing the importance of early referral for GHT in appropriate patients when growth failure or short stature is recognized. From the results of this analysis of the changes in mean HSDS and corrected HSDS from baseline to NAH, it would appear that these patients did experience a beneficial response to GH treatment. Sex was confirmed to be a statistically significant factor for NAH outcome, possibly due to the difference in referral age between sexes (females were younger than males). The significant negative correlation observed between ATS and near-adult HSDS for both sex subgroups indicates that, although a beneficial effect may be observed when GH therapy is initiated at an older childhood age, greater near-adult HSDS can be expected if these patients had started GHT at a younger age. Healthcare providers should therefore be aware of the potential limitations of this treatment among patients of a relatively advanced age. The correlation results of this study in relatively older children are consistent with recently published analyses of GH treatment on increasing linear growth to NAH or final height in younger children with IGHD or MPHD from other large patient databases [22–26]. This has also been shown in patients from other diagnostic groups, such as Noonan syndrome. Recently published ANSWER Program data on patients with Noonan syndrome with a mean referral age of 9 years showed that baseline age was negatively correlated with ΔHSDS after 1 or 2 years of GH treatment, indicating that older baseline ATS resulted in lower ΔHSDS [27–29]. In the present study, analysis of the relationship between age at initiation of GHT and near-adult HSDS in patients with GHD, ISS, and TS showed significant negative correlations, which is consistent with previous reports [13, 15, 28]. The results of the current analysis are among the first to describe the long-term effects of GH treatment, including an increase in HSDS at the time of NAH, in children who initiated GHT at an older age.

The effect of GH treatment on the BA/CA ratio was relatively small, and BA/CA remained <1 for patients across all indications over time. This suggests that, although GH treatment exerted positive effects on linear growth in this older pediatric patient population, there was a minimal effect on bone maturation relative to CA. The effect of pubertal stage in these patients was not analyzed and cannot be ruled out as a contributing factor. Also, the mean BA/CA ratio in the range of 0.82 to 0.88 indicates that, on average, a small amount of additional growth would have remained in some patients, consistent with near rather than final peak adult height.

Mean IGF-I SDS increased in patients with GHD, ISS, and TS at year 1 of GH treatment, remaining relatively stable thereafter. The difference in mean IGF-I SDS (GHD - ISS) was only -0.45 at baseline and 0.12 at NAH. From baseline to NAH, BMI SDS showed variable increases, but remained stable, during GHT among the diagnostic groups. This occurred in parallel with sustained increases in HSDS and corrected HSDS in each group during GH treatment. The negative mean BMI SDS at baseline observed in patients with ISS in the present study might suggest a degree of relative nutritional deficiency in these individuals that improved over time. Recent results published from the ANSWER Program registry indicated that for patients with GHD, a higher baseline BMI was positively correlated with the response to GH [13]. The association of BMI on GH response may reflect, in part, the importance of nutrition or nutritional status for optimizing outcomes in GH-treated patients [12, 13, 30, 31].

## Conclusions

In conclusion, long-term GH therapy in GH-naïve pediatric patients with GHD, ISS, or TS resulted in increases in HSDS and corrected HSDS from baseline to NAH despite their older age at the start of GHT. Although the results of this study demonstrate good NAH outcomes in children with short stature or growth failure who initiated GH treatment at an older age, the negative correlations of ATS with near-adult HSDS when analyzed by sex indicate that earlier recognition and referral for growth failure or short stature, and efforts to start GH treatment at a younger age, would most likely have resulted in greater gains in height and an increase in adult HSDS.

## Abbreviations

ATS: Age at treatment start
BA: Bone age
BMI: Body mass index
CA: Chronological age
GH: Growth hormone
GHD: Growth hormone deficiency
GHT: Growth hormone therapy
HSDS: Height standard deviation score
HV: Height velocity
IGF-I: Insulin-like growth factor-1
IGHD: Isolated idiopathic growth hormone deficiency
ISS: Idiopathic short stature
MPHD: Multiple pituitary hormone deficiency
NAH: Near-adult height
SDS: Standard deviation score
TS: Turner syndrome.
